# Characterization of the vitrification parameters for oviduct aggregates in *Camelus dromedarius*

**DOI:** 10.3389/fvets.2025.1561144

**Published:** 2025-07-02

**Authors:** Mohamed M. M. El-Sokary, Hamad A. Albreiki, Seham F. Shehata, Karima Gh. M. Mahmoud

**Affiliations:** ^1^Department of Theriogenology, Faculty of Veterinary Medicine, Benha University, Benha, Egypt; ^2^Faculty of Health Sciences, Higher Colleges of Technology, Abu Dhabi, United Arab Emirates; ^3^Department of Animal Wealth Development, Faculty of Veterinary Medicine, Benha University, Benha, Egypt; ^4^Department of Animal Reproduction and Artificial Insemination, Veterinary Research Institute, National Research Centre, Giza, Egypt

**Keywords:** isthmus, cryoprotectant, cryodevice, camel, reproductive biotechnology

## Abstract

**Introduction:**

Camel oviductal isthmus aggregates provide a novel and promising model for studying sperm attachment and longevity, offering a potential alternative for short-to mid-term sperm preservation and transport under non-cryogenic conditions. Effective cryopreservation of the aggregates for later use can contribute to addressing challenges associated with camel semen preservation by potentially extending sperm lifespan and facilitating semen transport to remote areas without cryogenic facilities. Challenges in preserving the structural integrity and viability of camel oviductal aggregates remain a key critical gap during cryopreservation. This study evaluated the efficiency of vitrification protocols for camel oviductal isthmus aggregates, focusing on the effects of aggregate size, cryoprotectants (CPA), cryodevices, post-thaw viability, and sperm-binding capacity.

**Methods:**

Aggregates retrieved from the oviductal isthmus were classified into four size groups (50, 100, 150, and 200 μm) and vitrified to determine the influence of size on post-thaw outcomes. CPA concentrations (3, 5, and 7 M) of DMSO and EG in a 1:1 ratio were tested for their impact on structural integrity and viability. The performance of cryodevices, including cryovials, 0.5 mL straws, and 0.25 mL straws, was also assessed.

**Results and discussion:**

The results indicated that aggregates sized 150 μm and 200 μm demonstrated superior post-thaw viability, with intactness rates of 78 ± 2.0% and 83 ± 2.8%, respectively. Among the tested CPA concentrations, 7 M showed the highest post-vitrification viability (69 ± 1.9%). Additionally, 0.25 mL straw cryodevice achieved significantly better post-thaw viability (67 ± 2.7%) compared to 0.5 mL straws (32 ± 2.1%) and cryovials (10 ± 1.1%). Regarding sperm-binding capacity post-thaw, aggregates treated with 5 M (69 sperm) and 7 M (74) CPAs showed the highest binding rates, with no significant difference between these concentrations. Further studies are required to optimize vitrification protocols to enhance the aggregate’s post-vitrification viability and structural integrity.

## 1 Introduction

The dromedary camel (*Camelus dromedarius*) holds significant cultural, economic, and recreational value in the Middle East, playing vital roles in milk and meat production, racing, and show competitions (1). Reproductive performance in this species remains a significant challenge, with fertility issues affecting both males and females. Low conception rates and male infertility are widespread concerns, limiting overall reproductive efficiency (2). One of the major limitations in advancing camel reproductive management is the incomplete understanding of the physiological mechanisms underlying fertility, particularly in the context of gamete interaction and embryo development (3). These challenges are further complicated by the difficulty of preserving camel semen, which is an essential component of assisted reproductive technologies. Maintaining semen viability and fertilizing capacity during extended storage remains a major obstacle, hindering the broader implementation of artificial insemination programs (4). Given these reproductive challenges, understanding the microenvironment where fertilization occurs is essential.

The oviduct, recognized as the site of fertilization, plays a critical role in ensuring reproductive success across various animal species. The oviduct exhibits a remarkable capacity to support sperm survival, particularly within the isthmus, or “sperm nest.” Sperm can be stored within the oviduct of swine and cattle for approximately 24 to 48 h, while in other vertebrate and invertebrate species, sperm storage may extend to several days or even months (5).

Oviductal cell aggregates have been adopted as an *in vitro* model to closely mimic the oviduct’s physiological environment and allow investigation of sperm behavior under conditions that resemble those *in vivo* (6). These aggregates are highly comparable to the *in vivo* oviduct in their three-dimensional architecture and microenvironmental features, including cell–cell interactions, secretory activity, and spatial organization. Such characteristics make them an ideal platform for exploring sperm attachment, motility, and survival (7). Supporting this approach, Dutta et al. (8) demonstrated that co-incubation of bovine sperm with bovine oviductal cell aggregates *in vitro* significantly extended sperm lifespan, emphasizing the potential of this model to preserve sperm viability.

However, despite these advances, a critical knowledge gap persists regarding the effective cryopreservation of camel oviductal isthmus aggregates, particularly how to preserve their structural integrity and biological functionality post-thaw to maintain their utility as a model for sperm preservation and fertility studies. To address this gap, the preservation of these cell aggregates has become an essential focus in cellular biology. Techniques adapted from stem cell and oncology research, such as the freezing of spheroids, have been employed to ensure the availability and readiness of these models for experimentation (9). By maintaining their three-dimensional structure and biological functionality post-preservation, these aggregates serve as a versatile and reliable tool for studying cellular behavior and advancing reproductive biology research.

In this context, the application and refinement of cryopreservation methodologies are essential to achieving successful preservation outcomes, particularly in the field of reproductive biotechnology. Cryopreservation is a cornerstone technique in reproductive and cellular biology, enabling the long-term storage and availability of biological materials such as cells, tissues, and aggregates for research and clinical applications (10). Various methods, including conventional freezing, programmable slow freezing, and vitrification, have been developed to preserve cellular integrity and functionality. Vitrification has emerged as a superior technique due to its ability to achieve higher cell survival rates (11). Among cryopreservation methods, vitrification has gained prominence due to its rapid cooling and improved cell survival. Given its advantages, vitrification represents a particularly promising approach for the cryopreservation of camel oviductal aggregates, providing a methodological basis for the current investigation on the success of cryopreservation.

Vitrification is an ultra-rapid cooling method that prevents ice crystal formation by solidifying cells into a glass-like state through direct exposure to liquid nitrogen and high concentrations of cryoprotectants (12). Unlike slow freezing, vitrification maintains cellular integrity by avoiding intracellular ice, which is particularly damaging to the delicate structure of mammalian oocytes (13). Its effectiveness is linked to the ability of high CPA concentrations to induce osmotic dehydration, minimizing cryo-injury during cooling and warming. Moreover, vitrification has become the method of choice in many species due to improved post-warming morphology and developmental potential.

The success of vitrification, however, depends on several critical factors that must be carefully optimized. These factors include the type, concentration, and permeability of the cryoprotectant agent (CPA) used, such as dimethyl sulfoxide (DMSO), glycerol, ethylene glycol, and propylene glycol, as well as the type of cryodevice (cryovials, straws, and cryotop) employed during the process (14). In addition to these parameters, the size and structural integrity of the aggregates are critical factors influencing cryosurvival, with careful handling and gentle pipetting significantly enhancing post-thaw recovery (15). Furthermore, the efficiency of a freezing protocol can be assessed through various methods, such as evaluating cell viability, membrane integrity, and mitochondrial function. Yet, beyond these immediate assessments, the ultimate measure of success lies in the ability of the frozen cells to resume their normal physiological capabilities after thawing, with minimal damage to their structure and function (16). These factors collectively determine the preservation of structural integrity and viability post-thaw.

Therefore, this study aimed to assess the efficacy of a vitrification protocol for camel oviductal isthmus aggregates by optimizing cryoprotectant concentration, evaluating the suitability of cryodevices, and determining the impact of aggregate size on post-thaw outcomes such as viability, structural integrity, and sperm-binding capacity, thereby supporting future research on sperm-oviduct interactions in camels.

## 2 Materials and methods

### 2.1 Ethical considerations

This study was conducted in accordance with ethical guidelines and approved by the Research Ethics and Integrity Committee at the Higher Colleges of Technology, under Approval Number REIC2024-FAC35. All experimental procedures adhered to institutional and international standards for the ethical use of biological materials in research and animal welfare.

### 2.2 Chemicals

All chemicals were purchased from Sigma-Aldrich unless otherwise stated.

### 2.3 Animals and sample collection

The study was conducted from September to November 2024. The samples for this study were collected from a local slaughterhouse in the Al Ain region, Abu Dhabi. Testes and their attached epididymides were obtained from 23 mature dromedary camels, aged between 5 and 10 years, at a local slaughterhouse. A total of 46 testes were collected over eight separate collections. Only testes that appeared normal in size, color, and consistency, with no visible lesions or abnormalities, were included in the study. Immediately following slaughter, the samples were placed in phosphate-buffered saline containing antibiotics (penicillin and streptomycin) and transported to the laboratory in a temperature-controlled container maintained at 4°C within 1 to 2 h.

### 2.4 Experimental design

This study was composed of five sequential experiments designed to systematically optimize key parameters influencing the cryopreservation of camel oviduct explant aggregates. The effects of aggregate size, cryoprotectant concentration, and cryodevice type on post-thaw viability, structural integrity, and reproductive functionality were evaluated. Each experiment built upon the findings of the previous one to enable stepwise refinement of vitrification conditions. Experiment 1 evaluated the impact of aggregate size on viability; Experiments 2 and 3 assessed the effects of cryoprotectant concentration on structural integrity and viability, respectively; Experiment 4 compared different cryodevices; and Experiment 5 examined the sperm-binding ability of thawed aggregates.

#### 2.4.1 Experiment 1: effect of aggregate size on post-thaw viability

This experiment aimed to identify the most suitable aggregate size for successful cryopreservation, hypothesizing that aggregate size influences cryosurvival due to factors such as cryoprotectant penetration and ice formation. Aggregates were categorized into four size groups: 50 μm, 100 μm, 150 μm, and 200 μm in diameter. Vitrification was performed using 0.25 mL straws. Post-thaw viability was assessed using SYBR14/PI dual staining, allowing differentiation between live and dead cells.

#### 2.4.2 Experiment 2: effect of CPA concentration on structural integrity

This experiment was conducted after determining the optimal aggregate size, focusing on optimizing CPA concentration to minimize cryo-injury and preserve the three-dimensional structure critical for aggregate function. The objective of this experiment was to evaluate the impact of varying CPA concentrations on the structural preservation of vitrified aggregates. Aggregates were exposed to three CPA concentrations—3 M, 5 M, and 7 M—consisting of dimethyl sulfoxide (DMSO) and ethylene glycol (EG) in a 1:1 ratio. Post-thaw structural integrity was analyzed to identify the concentration that best maintained aggregate morphology and cohesion.

#### 2.4.3 Experiment 3: effect of CPA concentration on post-thaw viability

This experiment aimed to establish the relationship between CPA concentration and cellular survival, determining the most suitable concentration for optimal viability. By evaluating viability alongside structural integrity, this step refined the CPA concentration choice to balance toxicity and cryoprotection. This experiment investigated the influence of CPA concentration (3 M, 5 M, and 7 M) on aggregate viability post-thaw. SYBR14/PI dual staining was performed to assess live and dead cell populations.

#### 2.4.4 Experiment 4: influence of cryodevice type on post-thaw viability

The fourth experiment was designed to compare the performance of different cryodevices in maintaining post-thaw aggregate viability. Three cryodevice types—cryovials, 0.5 mL straws, and 0.25 mL straws—were tested under identical vitrification and warming conditions. Post-thaw viability was evaluated to determine which cryodevice offered the most effective cryopreservation outcomes.

#### 2.4.5 Experiment 5: effect of CPA concentration on post-thaw sperm-binding ability

Based on the vitrification parameters identified in the previous experiments (the aggregate size, the CPA concentration, and the cryodevice), this experiment aimed to evaluate the impact of the CPA concentration on the capacity of oviduct aggregates to bind to sperm after thawing. Thawed aggregates were incubated with epididymal sperm, and the binding ability was assessed by quantifying the number of sperm bound to the aggregates. This experiment provided insights into how varying CPA concentrations influence the sperm-binding competence of vitrified aggregates post-thaw.

### 2.5 Retrieval of the epithelial cells from the oviduct

Oviducts were collected from apparently healthy pubertal female camels aged 4–8 years old (*n* = 10) slaughtered at a local abattoir and transported on ice to the laboratory at Higher Colleges of Technology, Abu Dhabi. The oviduct epithelial cells were collected and prepared at the laboratory according to El-Sokary et al. (7). Isolation of oviductal epithelial sheets from the isthmus involved gently squeezing them out with a glass slide angled at 45 degrees. The collected sheets were then transferred to a 15 mL conical tube and pelleted via centrifugation at 100 × g for 30 s. The supernatant was decanted, and the pellet was resuspended in 1 mL of capacitating TALP medium. Disaggregation was initiated by aspirating and expelling the suspension 10 times through a 1 mL pipette tip. The partially separated cells were then washed with 5 mL of fresh TALP and re-centrifuged at 100 × g for 1 min. After discarding the supernatant, the cells were re-suspended in an additional 1 mL of TALP. Complete disaggregation was achieved by passing the suspension through a 23-gauge needle 10 times. The total volume was then brought to 9 mL with TALP and equally aliquoted (3 mL each) into three 100-mm petri dishes and incubated at 39°C for 90 min to allow cells to re-aggregate.

### 2.6 Aggregate selection and size measurement

**Figure 1:**
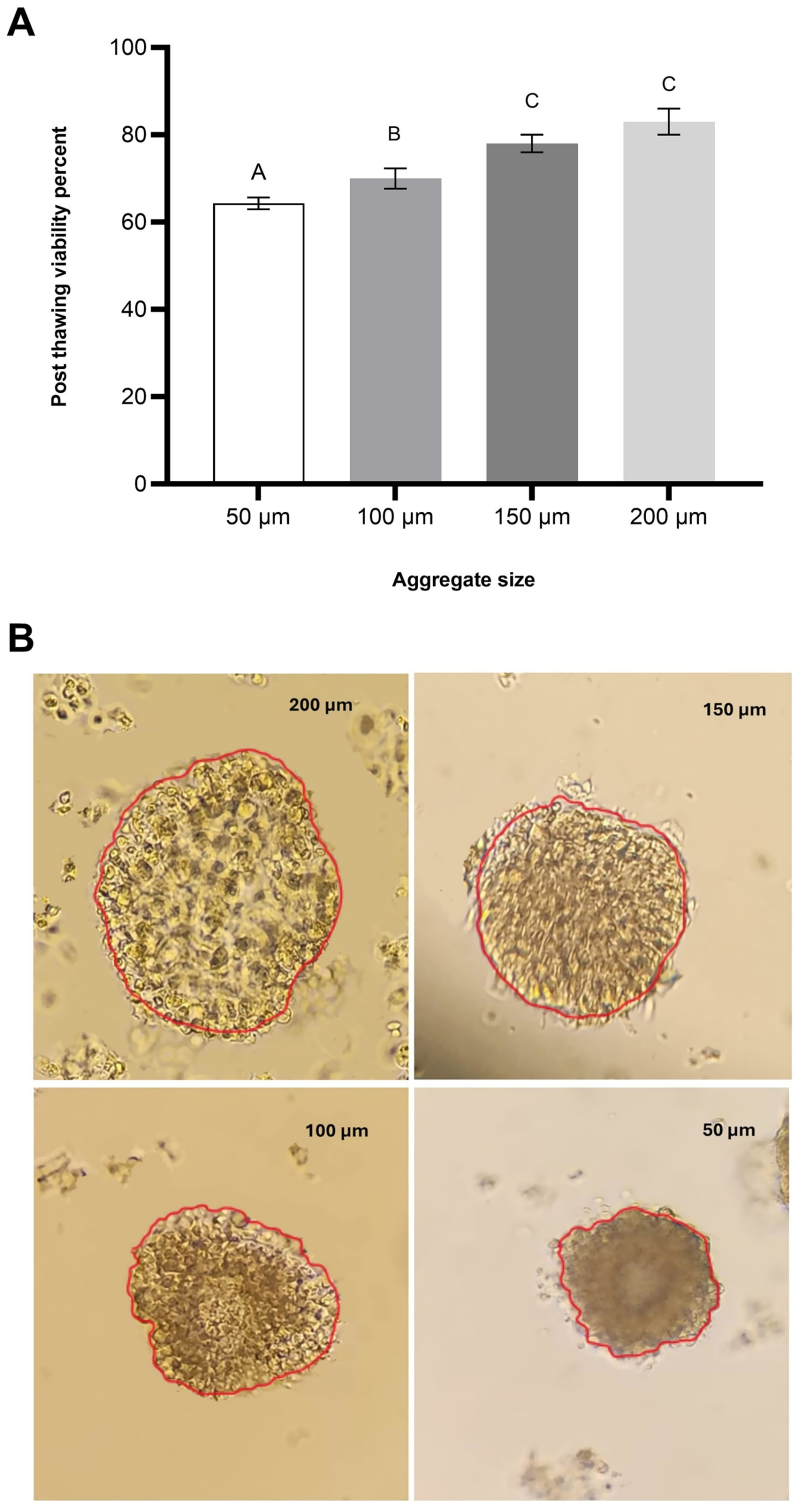
Effect of aggregate size on post-thaw viability of oviduct aggregates. **(A)** Column graph illustrating the post-thaw viability (%) of oviduct aggregates of varying sizes: 50 μm, 100 μm, 150 μm, and 200 μm. Columns with different letters denote statistically significant differences among the groups (*p* < 0.05). **(B)** Representative microscopic images of oviduct aggregates of different diameters (50 μm, 100 μm, 150 μm, and 200 μm). Scale bar = 50 μm.

After retrieval of oviduct tissue, aggregates were produced as described in the previous section. The resulting aggregates were washed three times by gentle pipetting in pre-warmed TALP medium to ensure their structural stability and viability during handling. Aggregates were then visually inspected under a stereo microscope (magnification: ×40–100) to identify and select those with clear, intact morphology (17). Aggregates were categorized into four size groups: 50 μm, 100 μm, 150 μm, and 200 μm in diameter (Figure 1B). To ensure accurate size measurement, aggregates were imaged using a digital microscope with calibrated imaging software (ImageJ). Diameters were measured across the widest part of the aggregates, and only those within the defined size ranges were included in the experiments. A total of 30 aggregates per size group were selected for observation in each experimental replicate.

### 2.7 Vitrification and warming by straw and cryovial methods

Oviduct aggregates were exposed to two-step cryoprotectant treatments at varying concentrations as illustrated in Table 1. The holding medium used during vitrification contained TCM 199 supplemented with 2.5 mM HEPES and 20% fetal calf serum. For cryovial vitrification, oviduct aggregates (*n* = 5–10) were transferred into 1.5 mL cryovials (Nunc, Rochester, MN, United States) and directly plunged into liquid nitrogen (LN2) (11). In the case of straw vitrification, the aggregates were immediately loaded into 0.25 mL or 0.5 mL straws, placed in the middle column of VS2, and separated by air bubbles. The straws were sealed with straw plugs and pre-cooled by exposure to liquid nitrogen (LN2) vapor at approximately 4 cm above the LN2 surface for at least 60 s before being vertically immersed in LN2 for storage for 2 weeks. Straws were transferred rapidly in less than 5 s through the air to avoid structural damage (18) and then placed in a water bath at 35–37°C for 20 s for warming. Following warming, the expelled oviduct aggregates were equilibrated for 5 min in a 0.5 M galactose solution (19) in TCM 199 to dilute and remove cryoprotectants. Oviduct aggregates were then washed 4–5 times in a fresh washing medium and cultured in a warm TCM medium for 2 h.

**Table 1:** Two-step cryoprotectant treatments applied to oviduct aggregates at varying concentrations.

CPA concentration	Step 1 (VS1)	Exposure time	Step 2 (VS2)	Exposure time
3 M	0.75 M EG + 0.75 M DMSO	45 s	1.5 M EG + 1.5 M DMSO	25 s
5 M	1.25 M EG + 1.25 M DMSO	45 s	2.5 M EG + 2.5 M DMSO	25 s
7 M	1.75 M EG + 1.75 M DMSO	45 s	3.5 M EG + 3.5 M DMSO	25 s

### 2.8 Assessment of the structural integrity of aggregates following vitrification

The structural integrity of the aggregates was evaluated post-vitrification and warming using phase-contrast microscopy (×100–200 magnification). Aggregates were assessed for compactness, shape, surface characteristics, and overall cohesion to determine their quality (20). The evaluation of the aggregates is categorized as shown in Table 2.

**Table 2:** Morphological grading criteria for post-thaw oviductal cell aggregates.

Grade	Description
Grade A (high quality)	Aggregates in this category exhibited a compact and rigid structure with no loose cells in the surrounding medium, reflecting strong cell adhesion and cohesion. The surface was smooth and intact, indicating healthy cellular connections. Morphologically, they maintained a spherical and uniform shape with consistent size, presenting a well-defined three-dimensional (3D) architecture that closely mimicked natural cellular interactions (Figures 4II,A)
Grade B (moderate quality)	Aggregates were generally compact and retained their spherical shape; however, minor surface irregularities and occasional deviations in size were observed. A few loose cells were present in the surrounding medium, indicating moderate cell cohesion and a slight reduction in overall quality (Figures 4II,B)
Grade C (low quality)	Aggregates displayed noticeable looseness with partially detached cells and irregular, inconsistent shapes. Variability in size and a lack of surface uniformity were also noted, reflecting compromised cell cohesion and structural integrity (Figures 4II,C)
Grade D (poor quality)	Aggregates exhibited severe structural disintegration, characterized by significant cell detachment and highly irregular, non-spherical shapes. The surface appeared rough and damaged, and the 3D architecture was severely disrupted, making them unsuitable for further analysis (Figures 4II,D)

### 2.9 Evaluation of viability in vitrified oviduct aggregates

**Figure 2:**
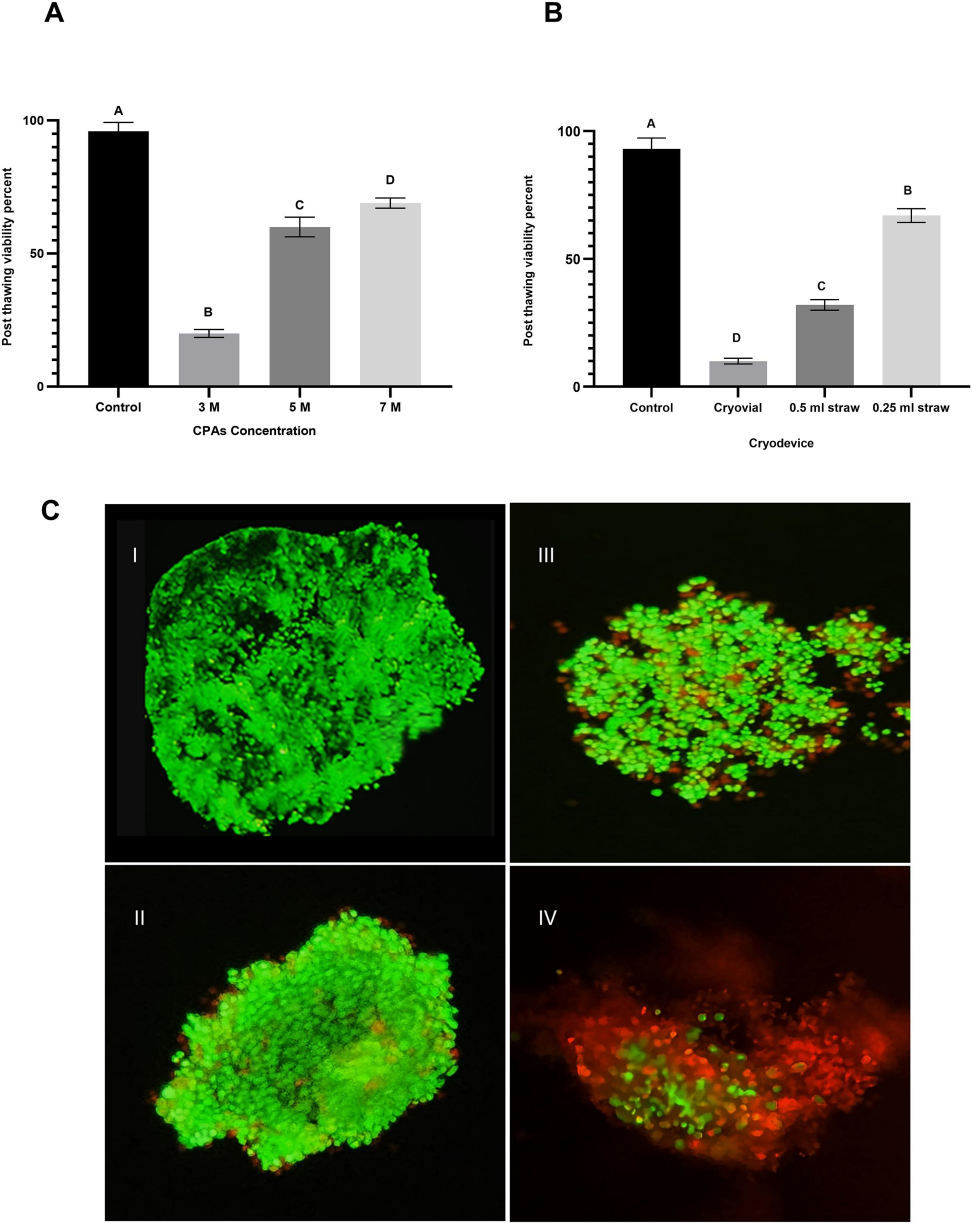
Impact of cryoprotectant (CPA) concentration and cryodevice type on post-thaw viability of oviduct aggregates. **(A)** Column graph illustrating the effect of CPA concentrations (3 M, 5 M, and 7 M) on post-thaw viability percentage of oviduct aggregates. Columns with different letters denote statistically significant differences among the groups (*p* < 0.05). **(B)** Column graph demonstrates the influence of cryodevice type (cryovials, midi-straws, and mini-straws) on post-thaw viability percentage. Columns with different letters denote statistically significant differences among the groups (*p* < 0.05). **(C)** Fluorescence microscopy images evaluating aggregate viability using SYBR-14 (green; live cells) and PI (red; dead cells) staining. **(C,I)** Control group. **(C,II)** 7 M CPA. **(C,III)** 5 M CPA. **(C,IV)** 3 M CPA. Scale bar = 50 μm.

To evaluate aggregate viability, SYBR-14 (1 μM) was added to the aggregate suspension and incubated for 10 min in a dark room to allow sufficient staining of the viable cells. Following this incubation, propidium iodide (PI, 1 μM) was introduced to the suspension and incubated for an additional 5 min. PI is a vital stain that can only penetrate the membranes of non-viable aggregates and emits a red fluorescence. In contrast, SYBR-14 stains the DNA of viable aggregates, producing a green fluorescence (21). The stained aggregates were then examined under a fluorescence microscope to visually differentiate between viable and non-viable aggregates based on the distinct green and red fluorescence patterns (Figures 2C,I–IV).

### 2.10 Epididymal sperm processing and preparation

Testes with epididymis were carefully separated and transported to the laboratory on chilled (approximately 4°C) physiological saline solution (0.9% NaCl) to minimize potential damage during transport. Upon arrival at the laboratory, the testes and epididymis were meticulously separated using sterile dissection techniques. The epididymides were then thoroughly rinsed with a sterile 0.9% saline solution to remove any residual blood or debris. Subsequently, the epididymal tissue underwent a brief dip in 70% ethyl alcohol for surface disinfection. The epididymis was incised to allow for the collection of sperm, which was subsequently examined [modified from Rashad et al. (22)]. The epididymal sperm was evaluated for motility and viability, and aliquoted for further use.

### 2.11 Assay of sperm binding to oviduct epithelial cells

**Figure 3:**
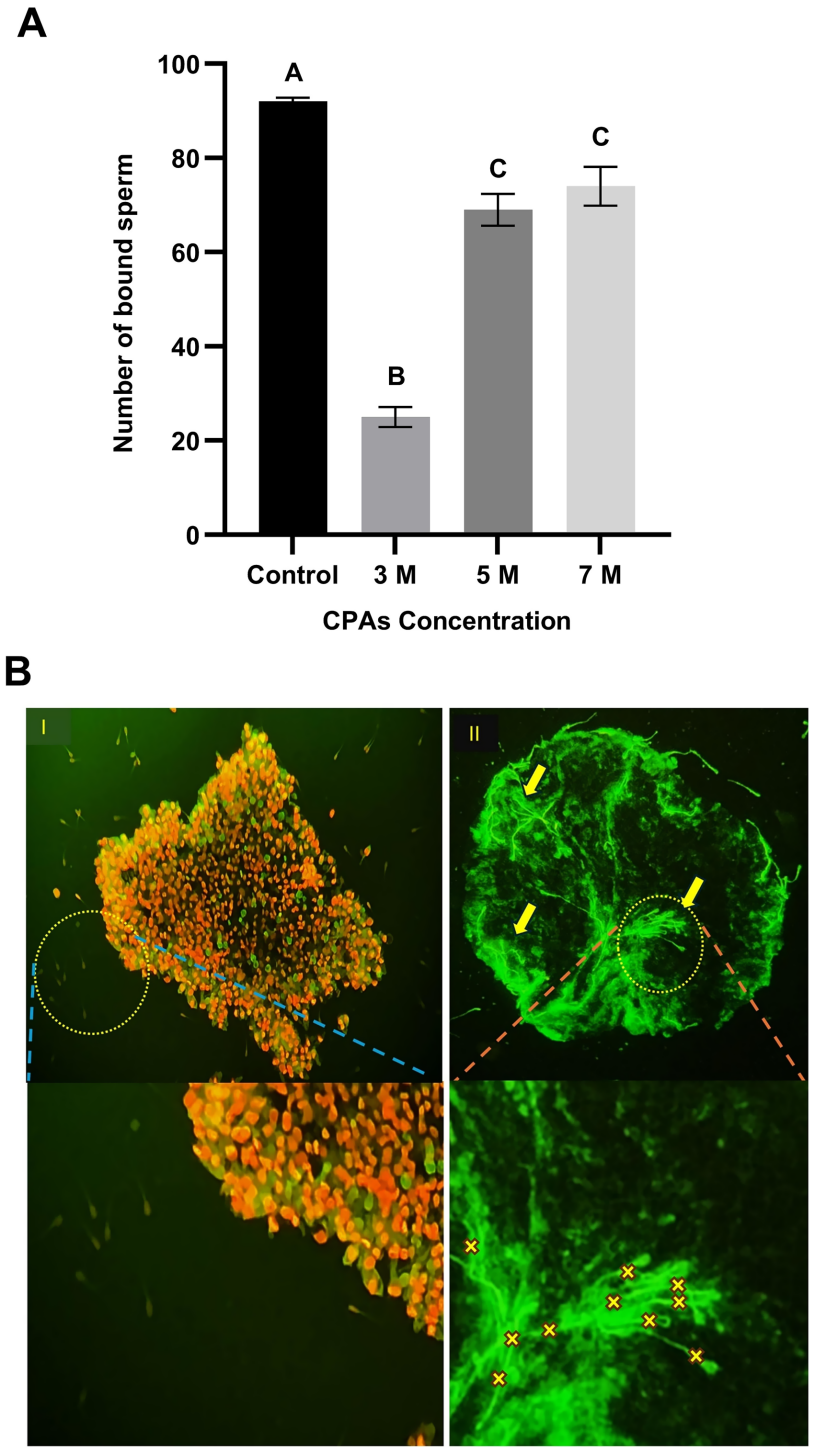
Effect of cryoprotectant (CPA) concentrations on sperm binding to post-thaw aggregates. **(A)** Number of sperm bound to oviduct aggregates following post-thawing across CPA concentrations (3 M, 5 M, and 7 M). Columns with different letters denote statistically significant differences (*p* < 0.05). **(B)** Fluorescence microscopy images illustrating sperm binding to oviduct aggregates. **(B,I)** Viable and intact aggregate post-thawing with sperm stained using MitoTracker Green. Yellow arrows and “X” indicate attached sperm. **(B,II)** Disintegrated aggregate post-thawing, with most cells stained with PI (red; dead) while few cells were stained with SYBR-14 (green; live), indicating extensive post-thaw damage and the absence of sperm binding. Scale bar = 50 μm.

The sperm-binding assay was carried out according to the methodology described by Winters et al. (17), with minor modifications as reported by El-Sokary et al. (7). Briefly, 20 oviduct cell explants were combined with 20 μL sperm droplets (1.6 × 10^6^ cells/mL) and the assay was performed in triplicate. Before incubation, sperm were stained with MitoTracker Green (1 μM) and allowed to stand for 10 min at room temperature in the dark room to ensure optimal staining. The stained sperm were then incubated with thawed oviduct cell aggregates at 39°C for 30 min. The experiment was independently replicated 3 times to ensure reproducibility. Each experimental condition utilized 30 oviductal aggregates, and following the incubation, unattached and loosely bound spermatozoa were carefully removed by washing with TALP medium. The number of spermatozoa bound to the periphery of each oviduct aggregate was subsequently quantified to estimate the binding capacity (Figures 3B,I,II).

### 2.12 Statistical analysis

Statistical analysis was performed separately for each experiment using one-way ANOVA to evaluate the effect of a single independent factor within each experiment (as in the experimental design). Tukey’s post-hoc test was used for multiple comparisons in each analysis, using IBM SPSS Statistics (version 22). A *p*-value of less than 0.05 was considered statistically significant. All experiments were independently repeated three times to confirm reproducibility. Data distribution was assessed for normality before analysis. Results are expressed as the mean ± standard error of the mean (SEM).

## 3 Results

### 3.1 Experiment 1: effect of aggregate size on post-thaw viability

The post-thaw viability of oviduct cell aggregates was significantly influenced by aggregate size. Aggregates with a diameter of 50 μm exhibited the lowest viability, with a mean value of 64.31 ± 0.33%. In the 100 μm group, viability increased to 70 ± 2.3% (*p* < 0.05). A further increase in aggregate size to 150 μm resulted in a higher viability of 78 ± 2.0% (*p* < 0.05), while the 200 μm group achieved the highest viability at 83 ± 2.8% (*p* < 0.05). No statistically significant difference was observed between the 150 μm and 200 μm groups (*p* > 0.05), indicating a plateau in post-thaw viability at these larger sizes (Figure 1A). However, both the 150 μm and 200 μm groups demonstrated significantly higher viability compared to the 100 μm group (*p* < 0.05), which itself was significantly higher than the 50 μm group (*p* < 0.05).

### 3.2 Experiment 2: effect of CPA concentration on structural integrity

**Figure 4:**
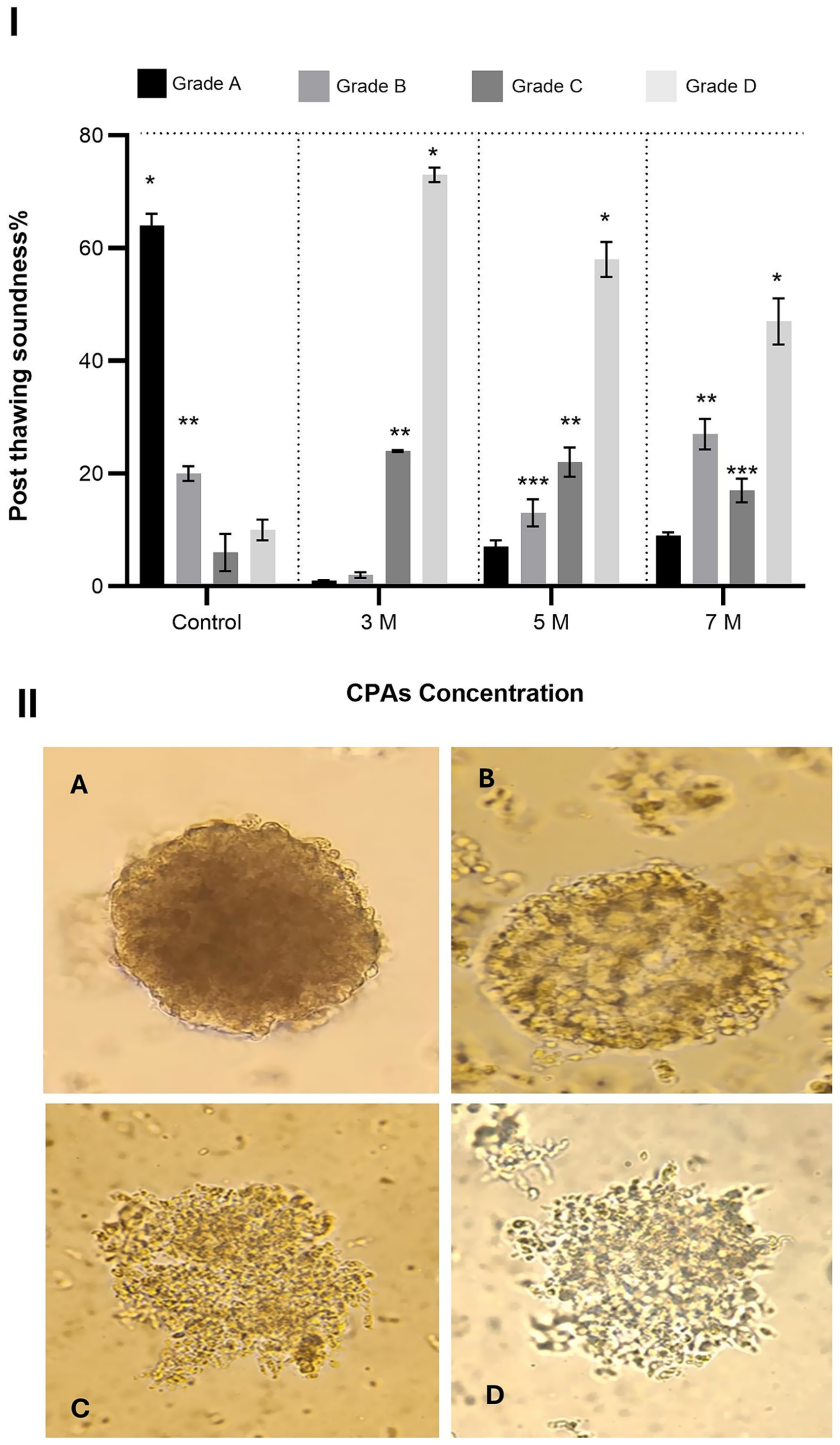
Effect of cryoprotectant (CPA) concentration on post-thaw structural integrity of oviduct aggregates. **(I)** Column graph showing the post-thaw soundness (%) of oviduct aggregates treated with CPA concentrations of 3 M, 5 M, and 7 M. Columns with asterisks denote statistically significant differences (*p* < 0.05). **(II)** Representative microscopic images illustrating different grades of aggregate structural integrity (Grades A to D). Scale bar = 50 μm.

The results of Experiment 2 are presented in Figure 4I. The concentration of cryoprotectant agents (CPA) significantly affected the post-thaw structural integrity of oviduct cell aggregates, as indicated by the distribution of Grades A, B, C, and D.

In the control group, Grade A aggregates were the most abundant (64 ± 2.1%), significantly higher than Grade B (20 ± 1.3%), Grade C (6 ± 3.3%), and Grade D (10 ± 1.8%) (*p* < 0.05). Grade B was also significantly higher than Grade C and Grade D (*p* < 0.05). When exposed to 3 M CPA, Grade A aggregates dropped sharply to 1 ± 0.13%, while Grade D aggregates increased substantially to 73 ± 1.3%, becoming the most dominant. Grades B and C were 2 ± 0.5% and 24 ± 0.19%, respectively. Grade D was significantly higher than Grade C, and Grade C was significantly higher than Grades A and B (*p* < 0.05).

At 5 M CPA, Grade A aggregates increased slightly to 7 ± 1.2%, while Grade D decreased to 58 ± 3.1%. Grades B and C were 13 ± 2.4% and 22 ± 2.6%, respectively. Grade D was significantly higher than all other grades. Grade C was significantly higher than Grade B, and Grade B was significantly higher than Grade A (*p* < 0.05). At 7 M CPA, Grade A increased further to 9 ± 0.57%, and Grade D decreased to 47 ± 4.1%. Grades C and B were 27 ± 2.7% and 17 ± 2.1%, respectively. Grade D remained significantly higher than all other grades. Grade C was significantly higher than Grade B, and Grade B was significantly higher than Grade A (*p* < 0.05).

### 3.3 Experiment 3: effect of CPA concentration on post-thaw viability

The results of Experiment 3 are presented in Figure 2A. The effect of CPA concentration on the post-thaw viability of oviduct cell aggregates was evaluated across four experimental groups: control, 3 M, 5 M, and 7 M. The 3 M group showed a dramatic reduction in viability to 20 ± 1.5% (*p* < 0.05 compared to all other groups. Viability significantly improved in the 5 M group, with 60 ± 3.7% of aggregates retaining their viability post-thaw (*p* < 0.05) compared to 3 M) demonstrating a marked enhancement with an increased CPA concentration. The 7 M group exhibited further significant improvement, reaching 69 ± 1.9% viability (*p* < 0.05) compared to 5 M.

### 3.4 Experiment 4: influence of cryodevice type on post-thaw viability

The results of Experiment 3 are shown in Figure 2B. Post-thaw viability of oviduct aggregates was significantly affected by the type of cryodevice used. In the control group, viability was high at 93 ± 4.3% (*p* < 0.05). Among the cryopreserved groups, aggregates frozen in cryovials had the lowest viability at 10 ± 1.1% (*p* < 0.05). Viability improved in the 0.5 mL straw group, reaching 32 ± 2.1%, which was significantly higher than in cryovials (*p* < 0.05). The highest viability among cryopreserved samples was observed in the 0.25 mL straw group, with 67 ± 2.7%, which was significantly greater than both the cryovial and 0.5 mL straw groups (*p* < 0.05).

### 3.5 Experiment 5: effect of CPA concentration on post-thawing sperm binding ability to oviduct aggregates

The effect of CPA concentration on post-thaw sperm-binding ability was evaluated across four experimental groups: control, 3 M, 5 M, and 7 M. In the control group, the number of sperm bound to the oviduct aggregates was highest, with an average of 92 bound sperm, significantly higher than all CPA-treated groups (*p* < 0.05). Among the CPA-treated groups, the 3 M concentration resulted in the lowest sperm-binding ability, with an average of 25 sperm bound per aggregate (*p* < 0.05). In contrast, the 5 M and 7 M concentrations demonstrated significantly higher sperm-binding abilities (compared to 3 M group), with averages of 69 and 74 bound sperm, respectively. However, no significant difference was observed between the 5 M and 7 M groups. Statistical analysis confirmed that both 5 M and 7 M concentrations were significantly higher than the 3 M group. These findings suggest that CPA concentration affects sperm-binding ability, with higher concentrations (5 M and 7 M) maintaining better binding efficiency compared to the 3 M group (Figure 3A).

## 4 Discussion

This study evaluated the effects of aggregate size, cryoprotectant concentration, and cryodevice type on the structural integrity, post-thaw viability, and sperm-aggregate binding ability of oviduct aggregates, contributing to a better understanding of factors influencing cryopreservation outcomes. The results demonstrated a significant influence of aggregate size on post-thaw viability, with viability improving as aggregate size increased up to 150 μm. However, the plateau observed between the 150 μm and 200 μm groups suggests that additional increases in size do not further enhance viability, potentially due to diffusion limitations of cryoprotectants or increased thermal gradients.

Furthermore, CPA concentration played a pivotal role in maintaining structural integrity post-thaw. As CPA concentration increased from 3 M to 7 M, a gradual improvement in the proportion of Grade A aggregates was observed. This indicates that higher CPA concentrations can mitigate osmotic and ice crystal damage by stabilizing cellular membranes and intracellular structures (23). However, the concurrent increase in Grade D aggregates across all CPA-treated groups highlights the potential cytotoxicity associated with cryoprotectants at suboptimal concentrations.

Cryoprotectants (CPAs), particularly at the high concentrations used in vitrification, can impact mammalian cells by affecting membrane integrity and cellular metabolism. Agents like dimethyl sulfoxide (DMSO) and ethylene glycol effectively prevent ice formation but may cause osmotic stress and oxidative damage if exposure is prolonged or not well controlled. Moreover, the evaluation of sperm-binding ability revealed a concentration-dependent effect of CPAs. The reduced binding observed at 3 M suggests compromised aggregate functionality. The improved viability observed at the 150 μm aggregate size underscores its importance in cryosurvival. These findings align with previous studies emphasizing the role of aggregate structure in preserving cellular integrity during cryopreservation (15).

The observed increase in Grade A aggregates with higher CPA concentrations, despite some cytotoxic effects, supports previous insights into cryoprotectant dynamics. These results are consistent with earlier findings on the balance between cryoprotective efficacy and cytotoxic effects of CPAs (24). Increasing CPA concentration from 3 M to 7 M improved post-thaw viability, reflecting better protection against ice crystallization and osmotic shock (25). Although viability in the 7 M group was slightly lower than controls, this is likely due to mild residual CPA toxicity rather than substantial harm. This underscores the delicate balance between achieving cryoprotection and minimizing cytotoxicity, highlighting the importance of optimizing CPA concentration and exposure duration to maintain cell survival and function (26). In this study, the combination of EG and DMSO appeared beneficial in improving post-thaw outcomes. However, previous studies reported mixed findings. Yadav et al. (13) found no advantage in combining DMSO with EG, and (27) reported similar results when using either 20% EG or 20% DMSO individually.

The superior viability associated with the 0.25 mL straws highlights how cryodevice choice shapes post-thaw outcomes. The higher viability observed in 0.25 mL straws may be attributed to their ability to facilitate faster and more uniform cooling and warming rates, which are critical for minimizing intracellular ice formation and osmotic stress (28). Conversely, the lower viability in cryovials (1.5 mL) is likely due to their larger volume and slower cooling rates, which can exacerbate cryo-induced damage (29). The findings emphasize the importance of cryodevice selection in cryopreservation protocols, particularly for sensitive biological materials like oviduct aggregates. However, vitrification in 0.25-ml straws causes a delay in heat loss from the solutions, possibly leading to devitrification, i.e., intracellular recrystallization during warming (30). This apparent contradiction may be due to differences in sample composition, cryoprotectant penetration, or technical variations in the handling and warming steps.

The CPA concentration-dependent improvement in sperm binding reinforces the link between structural integrity and functionality. This is consistent with Parnpai et al. (16), who reported that a strong indicator of cryopreservation success is the ability of thawed cells to restore normal physiological function with minimal structural damage. The reduced binding rate is likely due to structural and membrane damage or alterations in the surface receptors essential for sperm interaction. The improved binding at 5 M and 7 M highlights the protective effects of optimal CPA concentrations in preserving the structural integrity of oviduct aggregates. However, the lack of significant difference between these two concentrations indicates a plateau in functional recovery, which mirrors the trends observed in viability. These findings highlight the intricate balance between maintaining structural integrity and preserving functional capabilities during cryopreservation (31).

Efficient vitrification and recovery of the aggregates may support the development of more refined *in vitro* fertilization protocols, improve sperm selection strategies, and serve as biological carriers for oviductal secretions that influence fertilization (32). The successful vitrification protocol of the aggregates offers different practical applications, including but not limited to their importance as a promising tool for studying sperm-oviduct interactions under near-physiological conditions, particularly in species like camels, where *in vivo* studies are limited.

## 5 Conclusion

In conclusion, this study demonstrates that aggregate size, cryoprotectant concentration, and cryodevice type significantly affect post-thaw viability, structural integrity, and sperm-binding ability of oviduct cell aggregates. Specifically, increasing aggregate size up to 150 μm improves viability, with no further benefit observed at 200 μm. Cryoprotectant concentration exhibits a clear dose-dependent effect: low levels (3 M) result in poor viability and structural damage, whereas higher concentrations (7 M) substantially enhance viability and preserve sperm-binding function. Among the cryodevices tested, the 0.25 mL straw outperforms both cryovials and 0.5 mL straws, achieving the highest post-thaw viability. Based on our findings, we recommend cryopreserving oviduct cell aggregates approximately 150 μm in diameter using a 7 M cryoprotectant concentration and 0.25 mL straw cryodevices to achieve optimal preservation outcomes. This optimized protocol offers a reliable model for the cryopreservation of oviduct aggregates, with promising applications in reproductive biology research and fertility preservation.

## Data Availability

The raw data supporting the conclusions of this article will be made available by the authors, without undue reservation.
